# Functional near-infrared spectroscopy imaging of the prefrontal cortex during a naturalistic comedy movie

**DOI:** 10.3389/fnins.2022.913540

**Published:** 2022-09-08

**Authors:** Noam Somech, Tamar Mizrahi, Yael Caspi, Vadim Axelrod

**Affiliations:** ^1^The Gonda Multidisciplinary Brain Research Center, Bar-Ilan University, Ramat Gan, Israel; ^2^Head Injuries Rehabilitation Department, Sheba Medical Center, Ramat Gan, Israel; ^3^Department of Psychology, Bar-Ilan University, Ramat Gan, Israel

**Keywords:** functional near-infrared spectroscopy (fNIRS), naturalistic stimuli, movie, humor, intersubject correlation (ISC), prefrontal cortex

## Abstract

Naturalistic stimulation (i.e., movies and auditory narratives of some minutes’ length) has been a powerful approach to bringing more real-life experiences into laboratory experiments. Data-driven, intersubject correlation (ISC) analysis permits examining to what extent activity in a specific brain region correlates across participants during exposure to a naturalistic stimulus, as well as testing whether neural activity correlates with behavioral measures. Notably, most of the previous research with naturalistic stimuli was conducted using functional fMRI (fMRI). Here, we tested whether a naturalistic approach and the ISC are feasible using functional near-infrared spectroscopy (fNIRS) - the imaging method particularly suited for populations of patients and children. Fifty-three healthy adult participants watched twice a 3-min segment of a Charlie Chaplin movie while we recorded the brain activity on the surface of their prefrontal cortex using fNIRS. In addition, an independent group of 18 participants used a continuous scoring procedure to rate the extent to which they felt that different parts of the movie fragment were funny. Our two findings were as follows. First, we found higher-than-zero ISC in fNIRS signals in the prefrontal cortex lobes, a result that was particularly high in the oxygenated channels during the first repetition of the movie. Second, we found a significant negative correlation between oxygenated brain signals and ratings of the movie’s humorousness. In a series of control analyses we demonstrated that this latter correlation could not be explained by various non-humor-related movie sensory properties (e.g., auditory volume and image brightness). The key overall outcome of the present study is that fNIRS in combination with the naturalistic paradigms and the ISC might be a sensitive and powerful research method to explore cognitive processing. Our results also suggest a potential role of the prefrontal cortex in humor appreciation.

## Introduction

Cognitive neuroscience experiments have traditionally been conducted in laboratory settings for two obvious reasons. First, most neuroimaging experiments e.g., those using functional fMRI (fMRI)] cannot feasibly be conducted outside the laboratory. Second, the laboratory setup permits designing a relatively controllable experiment. The drawback of the laboratory setup is that the participants’ experience is usually far from real-life (Neisser, 1976; Holleman et al., 2020). Current technological advancement permits conducting experiments in fully naturalistic environments like walking on a street (Pinti et al., 2018), but such technologies are still associated with major technical challenges (e.g., motion artifacts). To this extent, a more controllable and successful approach to bringing real-life experience into laboratory experiments has been the use of movies or auditory stories, naturalistic stimuli of long duration (e.g., minutes or even an hour) - the approach pioneered by Hasson et al. (2004) as well as Bartels and Zeki (2004).

The data-driven intersubject correlation (ISC) is a widely used approach to analyze experiments with naturalistic stimuli (Nastase et al., 2019). The essence of this approach is to test whether neural activity in a specific brain region of interest during a stimulus (e.g., watching a movie) is correlated (i.e., synchronized) across participants. If so, this implies that activity in this region of interest was at least to some extent related to (or driven by) naturalistic stimulus. That is, without a stimulus, as in the case of spontaneous, resting-state activity (Raichle and Snyder, 2007), no synchronization in neural activity among participants is expected. ISC during movies and auditory narratives has been shown as reliable (Hasson et al., 2010; Simony and Chang, 2020) not only in sensory regions (Hasson et al., 2004; Kauppi et al., 2010; Cantlon and Li, 2013; Lahnakoski et al., 2017), but also in the high-level prefrontal cortex (Jääskeläinen et al., 2008; Wilson et al., 2008; Kauppi et al., 2010; Lerner et al., 2011; Honey et al., 2012a; Simony et al., 2016). Naturalistic stimuli and ISC have helped to address a wide range of cognitive questions that are either too complicated or impossible to address using other methods. For example, using naturalistic stimuli, the researchers investigated the length of windows for information integration (Hasson et al., 2015; Chen et al., 2016) and natural stimuli event boundaries (Baldassano et al., 2017; Ben-Yakov and Henson, 2018). By providing the participants with two different contextual cues before the auditory story, it was possible to explore mechanisms of real-life context processing (Yeshurun et al., 2017). Similarly, it was possible to investigate how individual personality traits modulate processing of the auditory story (Finn et al., 2018). By first showing the participants a movie and then asking them to recall the content, Chen et al. (2017) demonstrated similarity between perceptional and memory processes. Naturalistic stimuli and the ISC data-driven approach have been also instrumental in investigation of social cognition, a field in which it is difficult to generate *a priori* hypotheses because the stimulus space is high-dimensional (Adolphs et al., 2016; Nummenmaa et al., 2018). For example, Richardson et al. (2018) demonstrated how activity in the brain networks responsible for theory of mind and pain changes in the course of development. Finally, naturalistic stimuli and ISC have also been successfully used to diagnose high-level cognition and consciousness in disorder of consciousness patients (Naci et al., 2014, 2017). The naturalistic stimuli and ISC approach have been most widely applied using fMRI (for review: Nastase et al., 2019) and to a lesser extent using electroencephalography (EEG; Chang et al., 2015; Ki et al., 2016; Poulsen et al., 2017; Laforge et al., 2020), magnetoencephalography (MEG; Betti et al., 2013; Lankinen et al., 2014; Chang et al., 2015; Puschmann et al., 2021), or intracranial recordings (Mukamel et al., 2005; Honey et al., 2012b). However, functional near-infrared spectroscopy (fNIRS) - the method used in the present study – was not previously used except for one study discussed below (Rowland et al., 2018).

Functional near-infrared spectroscopy is a rapidly developing and burgeoning non-invasive neuroimaging method (Pinti et al., 2020). By shining and collecting near-infrared light on the surface of the head, fNIRS allows measurement of changes in oxygenated (HbO) and deoxygenated hemoglobin (HbR) - the signals which reflect neural activity (Hoshi, 2016). fNIRS and fMRI are both hemodynamic-based imaging methods (Scarapicchia et al., 2017). Thus, given that the naturalistic approach was successful in fMRI studies, the question is whether fNIRS is also sensitive to this type of paradigm. This inquiry is important not only from a theoretical but also from a practical point of view because fNIRS with naturalistic paradigms is particularly suitable for clinical populations and children. Naturalistic paradigms are engaging and relatively effortless, a valuable aspect in experiments with clinical populations (e.g., Naci et al., 2014) and children (Davidson et al., 2003; Vanderwal et al., 2015). fNIRS is a portable device and is therefore well suited for testing a clinical population, especially at the bedside (Rupawala et al., 2018; Chen W. L. et al., 2020). The use of fMRI with children is associated with many challenges (Barkovich et al., 2019; Copeland et al., 2021); therefore, the use of fNIRS with children, at least in some contexts, might be advantageous (Yeung, 2021). To the best of our knowledge there has been only one study to date that has employed fNIRS with naturalistic stimuli and ISC (Rowland et al., 2018). Using auditory narratives, Rowland et al. (2018) found significant ISC correlation in several channels of the auditory cortex as well as in the prefrontal cortex. However, the study did not find a significant correlation between fNIRS signals and self-reported behavioral measures (i.e., listening effort). Thus, while this study provides initial evidence, more investigation regarding the sensitivity of fNIRS to naturalistic stimuli is warranted.

**FIGURE 1:**
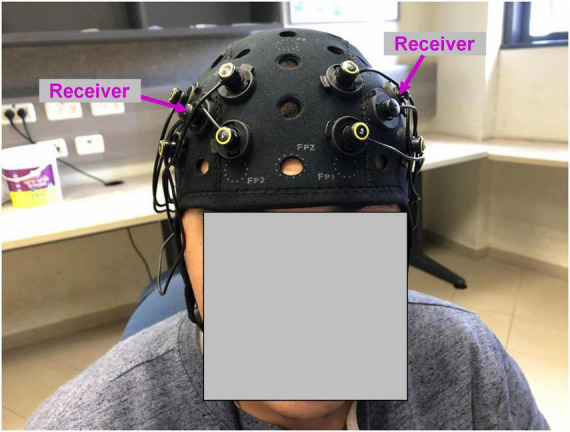
Functional near-infrared spectroscopy montage used in the study. Four emitters in each hemisphere were positioned at the corners of the square around the receiver (marked with magenta arrows). The position of the receiver was AF3 (left hemisphere) and AF4 (right hemisphere) according to 10–20 EEG system.

The central goal of the present study was to test the feasibility of using fNIRS with naturalistic paradigms. Beyond examining whether naturalistic stimulus elicits synchronous activity across participants (ISC), we were interested in testing a correlation between neural activity and behavioral response - the type of analysis that might be more informative with regard to cognitive functions of interest. In our study, we examined humor processing - a high-level cognitive capacity which plays an important role in our lives (Martin and Ford, 2018). The mechanisms of humor processing are not fully understood, while at the neural level a wide range of regions was associated with humor processing (Vrticka et al., 2013). To this extent, the lateral prefrontal cortex is particularly interesting. On the one hand, some studies reported increased activity in the lateral prefrontal cortex during humor appreciation (Goel and Dolan, 2001; Mobbs et al., 2005; Neely et al., 2012), but according to an alternative view, in a situation without humor, the prefrontal cortex might actively inhibit humor-related behavior such as laughter (Wild et al., 2006). Accordingly, during humorous content, the prefrontal cortex might be deactivated (Wild et al., 2006; Adamczyk et al., 2017). To shed light on this question, we focused on the prefrontal cortex in our study. It is noteworthy that the fMRI ISC effects in the prefrontal cortex have been weaker compared to the effects in the sensory regions (Hasson et al., 2004; Lerner et al., 2011); therefore, testing the frontal lobes is also a more difficult test-case scenario with regard to the feasibility of using fNIRS with naturalistic stimuli and ISC. Fifty-three healthy volunteers watched twice a 3-min fragment from Charlie Chaplin’s *Circus* (1929). In parallel to watching the movie, bilateral activity of the prefrontal cortex was recorded using fNIRS (Figure 1), permitting us to examine the ISC of prefrontal activity. In addition, a separate and independent group of 18 participants rated the same movie as to what extent each point of the movie fragment was funny.

## Materials and methods

### Functional near-infrared spectroscopy experiment

#### Participants

Fifty-three healthy participants took part in the fNIRS experiment (average age: 24.8, range: 18-41, standard deviation (SD): 4.61, 34 males, 4 left-handed). The planned analysis was the group-level comparison of correlation (ISC) values vs. zero (i.e., one-sample, two-tailed *t*-test vs. zero). Accordingly, the sample size we used was an approximate sample needed to achieve a moderate effect (Cohen’s *d* = 0.5) in this analysis (Faul et al., 2007). Note, that our sample was much larger than was previously used in the ISC fMRI studies (Jääskeläinen et al., 2016; Pollick et al., 2018; Adebimpe et al., 2019; Leong et al., 2020; Sachs et al., 2020; Hu et al., 2022) and one fNIRS study (Rowland et al., 2018). In addition, for the fMRI data, it has been shown that the sample size of 20 participants was sufficient for high reliability of the effects in the ISC analysis (Pajula and Tohka, 2016). The study was approved by the Gonda Multidisciplinary Brain Research Center ethics committee at Bar-Ilan University, Israel. The experiment was executed in line with full ethical approval. Prior to participating in the experiment, informed consent forms were signed by the participants. For taking part in the experiment, participants received monetary compensation or credit points.

#### Stimuli and procedure

We used a 3-min fragment of *The Circus* (1928), a silent movie with Charlie Chaplin as director and principal actor. In this fragment, the protagonist (i.e., a Charlie Chaplin character) spends time in a cage with a lion after locking himself inside by mistake. The fragment includes various comic episodes that happen to the protagonist. Participants watched the movie on a 24” monitor (full-screen mode), while they were sitting about 50 cm from the screen. The sound was delivered *via* headphones. The 3-min movie fragment was replayed twice. Participants were not recorded on camera during the experiment. After watching both repetitions, participants answered a behavioral questionnaire. The first two multiple-choice questions focused on understanding the movie’s content. The two additional questions [Likert scale (1: minimum; 5: maximum)] verified the experiences of the participants while watching the movie. The first question was “To what extent did you have thoughts unrelated to the movie (e.g., personal thoughts)?” The second question was “To what extent did you like the movie?”

The design of our experiment was guided by the following principles: we wanted to present the same movie twice in order to test the reproducibility of the fNIRS signal across two repetitions (for a similar design: Jääskeläinen et al., 2016). The duration of each repetition was 3 min, which is shorter than has been previously used in fMRI studies. In fNIRS the sampling rate is much higher than in the fMRI (Geng et al., 2017); therefore, we hypothesized that the 3-min length might be sufficient to establish reliable ISC. In addition, the 3-min fragment was a self-contained comedy episode (i.e., Charlie Chaplin in the cage with a lion) that we also used for rating humorousness and subsequent correlation with fNIRS activity. The movie did not contain additional similar episodes with regard to humor (i.e., a similar style of humor); thus, having a longer movie would have been problematic with regard to fNIRS vs. humorousness rating correlation.

#### Apparatus and functional near-infrared spectroscopy imaging

While watching the movie, neural recording was conducted using OctaMon fNIRS device (Artinis, Netherlands). The recording system included eight light emitters and two light receivers, resulting in eight HbO and eight HbR channels. Four emitters and one receiver were positioned bilaterally and symmetrically on the surface of the prefrontal cortex (Figure 1). The position of the receivers corresponded to AF3 (left hemisphere) and AF4 (right hemisphere) according to 10–20 EEG system. The emitters were positioned at the corners of the square around the receiver. The distance between emitters was 30 mm. To measure oxyhemoglobin (HbO) and deoxyhemoglobin (HbR) signals, continuous near-infrared light of 758 and 843 nm was used. The sampling rate was 10 Hz.

### Behavioral experiment for rating humorousness of the movie

#### Participants

An independent group of twenty participants took part in the experiment that included watching a movie and then rating its humorousness (average age: 30.7, range: 24-50, SD: 7.9, 11 males, all right-handed). Three participants were excluded from the analysis because they did not follow the experiment’s instructions. The study was approved by the Gonda Multidisciplinary Brain Research Center ethics committee at Bar-Ilan University, Israel. The experiment was executed in line with ethical approval. Prior to participating in the experiment, informed consent forms were signed by the participants. For taking part in the experiment, participants received monetary compensation.

#### Stimuli and procedure

The stimulus and the watching procedure were the same as discussed for the fNIRS experiment. The only difference was that the movie fragment was repeated only once. After watching the movie fragment, participants continuously rated the movie fragment with regard to how funny each point was [Likert scale 1 (minimal) - 5 (maximal)]. Collecting ratings of the movie’s humorousness is a common practice (Hutcherson et al., 2005; Watson et al., 2006; Franklin and Adams, 2011; Jääskeläinen et al., 2016). We created a custom MATLAB user interface to rate the humorousness of the movie. Specifically, the static images of the movie were extracted and were loaded into the MATLAB interface. While the original movie frame rate was 25 frames per second, in the rating procedure we used each fifth frame (i.e., 5 frames per second rate). Rating each single frame was redundant because the movie plot did not change every 40 ms. In total, the participants had to rate 900 frames (180 s × 5 frames per second). Upon issuing a rating, the next frame appeared automatically. The rating procedure was conducted without movie sound. We instructed the participants to base their ratings on impressions they had while watching the movie. Our ratings were more frequent, compared for example to a rating each 15 s used by Jääskeläinen et al. (2016) in the fMRI study. We decided to use a relatively high sampling rate for humorousness ratings because the sampling rate of fNIRS is relatively high (at least, compared to the sampling rate of the fMRI). As a result, both neural and behavioral time-courses had high sampling rates. Dense ratings of humorousness also ensured that no relevant scene was missed. By performing the rating frame by frame, the participants had the impression they were slowly watching a movie.

### Data analysis (both experiments)

In the fNIRS experiment, the values of HbO and HbR were obtained using the modified Beer-Lambert law (Artinis, Oxysoft software). Data analysis was performed in MATLAB using home-made code (Axelrod, 2014). Preprocessing included standard steps (Dans et al., 2021; Yücel et al., 2021) executed in the following order: Gaussian temporal smoothing (Schwartz et al., 2021) (FWHM = 200 ms), detrending (MATLAB detrend function), and band-pass second-order Butterworth filter (0.01-0.5 Hz) (Wiggins et al., 2016). After that, the time-courses were standardized using the *z*-score Matlab function. Temporal smoothing and band-pass filtering were intended to remove physiological vasomotor regulations and breathing-related signal fluctuations (Pinti et al., 2019). However, these steps did not remove artifacts due to the motion of the participants; [for example, see Fig. 1 in Cooper et al. (2012)]. To address the latter, we adopted a motion artifact rejection procedure that was similar to the hmrMotionArtifact function of the HOMER2 package (Huppert et al., 2009). Specifically, for each channel we first identified the local peak samples, which were three or more SDs beyond or below average. The samples from both sides of the peak up to the level of one SD from the mean with an additional 500 ms (five samples) margins from both sides were discarded. Compared to removal only of those samples beyond and below a SD-based threshold, our procedure ensured a more complete removal of the motion artifact. The average number of excluded time-points (i.e., samples) per channel was 4.2% (SD: 0.03%). The time-courses at the end of preprocessing were visually inspected and validated.

Our analyses were conducted for fNIRS signals averaged across four channels within the hemisphere, resulting in four signals referred to below as channels (bilateral HbO and HbR). The ISC was conducted using two commonly used types of analysis: leave-one-subject-out and pair-wise approach (Nastase et al., 2019). In the leave-one-subject-out approach, for each of four channels the time-courses of N-1 participants (52) were averaged, and the resultant time-course was correlated with the time-course of the remaining participant. This procedure was repeated 53 times, yielding a correlation value for each participant and channel. The average time-course of N-1 participants was calculated by omitting the segments of data excluded due to motion. In the other words, the averaged channel time-course in each time-point was calculated for all subjects except for those with an omitted sample in this specific time-point. In the pair-wise analysis, for each channel, all pair-wise correlations between time-courses of the participants were conducted. In both analyses, the correlation was calculated without the segments of data excluded due to motion (i.e., only the time-points that were present in both time-courses were used). To ensure normality of the distributions, Fisher *z*-transformation was applied to the ISC values (Nastase et al., 2019).

To establish the significance of the effect, for each channel and repetition of the movie, we used one-sample *t*-test as well as a permutation non-parametric approach with a random shift of the time-course (Nastase et al., 2019). The implementation of the permutation analysis for the leave-one-subject-out approach was as follows (described for one channel). First, we generated a vector of random shifts of the time-course (10,000 values; minimal shift: 0 points, maximal shift: number of samples−1). Second, for each participant (i.e., a participant vs. N-1 average correlation), we repeated the correlation analysis 10,000 times by circularly shifting one time-course each time by the predefined number of samples (see previous item). Third, the correlation values for each shift were averaged across participants, generating a synthetic distribution. Fourth, the number of correlation values in averaged synthetic distribution larger than the across-subject average of non-permutated correlation values was calculated. Fifth, to obtain the *p*-value, the results from previous items were multiplied by 2 (to have a two-tailed test) and divided by 10,000. The implementation of the permutation analysis for the pair-wise approach was similar to the one described above, with the only difference being that in the second stage, the analysis was repeated for each pair of time-courses. The circular shift permutation analysis is a more conservative approach than a bootstrap correlation analysis (Wilcox, 2012; Axelrod et al., 2017). The advantage of circular shift permutation analysis is that it preserves the original time-course while only the relative position of two time-courses changes. In general, it is preferable that the data used in permutation analysis should be as similar as possible to the real data. To test whether lower ISC during the first repetition of the movie can be potentially explained by the larger motion artifacts of the participants, during the second repetition, for each channel and participant we compared the number of excluded samples during preprocessing for the first and second repetitions of the movie. All our analyses in the paper were conducted for the channels, which were averages of the four channels. Accordingly, the number of excluded frames was also an average across the four channels. The results of this analysis were qualitatively similar when conducted for individual channels.

For the correlation analysis between time-courses (Figures 3, 4), the fNIRS and humorousness ratings were averaged across the participants. The sampling rate of the humorousness ratings and fNIRS data were 5 and 10 samples per second, respectively. Accordingly, for this analysis we down-sampled fNIRS data to a 5 samples per second rate by averaging each two consecutive data-samples. To calculate the significance of the correlation between time-courses, as in the previous analysis, we used a permutation analysis (10,000 permutations) with a circular shift of the time-course by a random number of samples. Specifically, similar to the implementation described above, one of the time-courses was circularly shifted by a random number of samples, and the correlation was calculated. This procedure was repeated 10,000 times, resulting in a synthetic distribution. To test the correlation between humorousness ratings and subject motion (i.e., to examine whether participants had greater motion during funnier movie segments), for each subject and channel, we determined whether the sample at each time-pointed was excluded at the preprocessing stage. This resulted in a “motion time-course” per each channel and participant. Then, the time-courses across participants were averaged. Significance of the correlation between humorousness ratings and averaged “motion time-course” was established using circular shift permutation analysis, as described above. To test the specificity of the correlation between fNIRS neural signals and humorousness ratings, we examined correlations of fNIRS neural signals with four non-humor-related features of the movie. Specifically, the properties were as follows. First, we extracted the auditory sound volume of the movie. Second, for each movie frame, we calculated mean gray-scaled brightness. Third, as an estimate of visual motion within the movie, we calculated changes in pixel brightness in consecutive frames. Fourth, to estimate the complexity of the visual scene following the approach used previously (Chai et al., 2010) for each movie frame, we calculated number of objects (both inanimate and animate).

**FIGURE 2:**
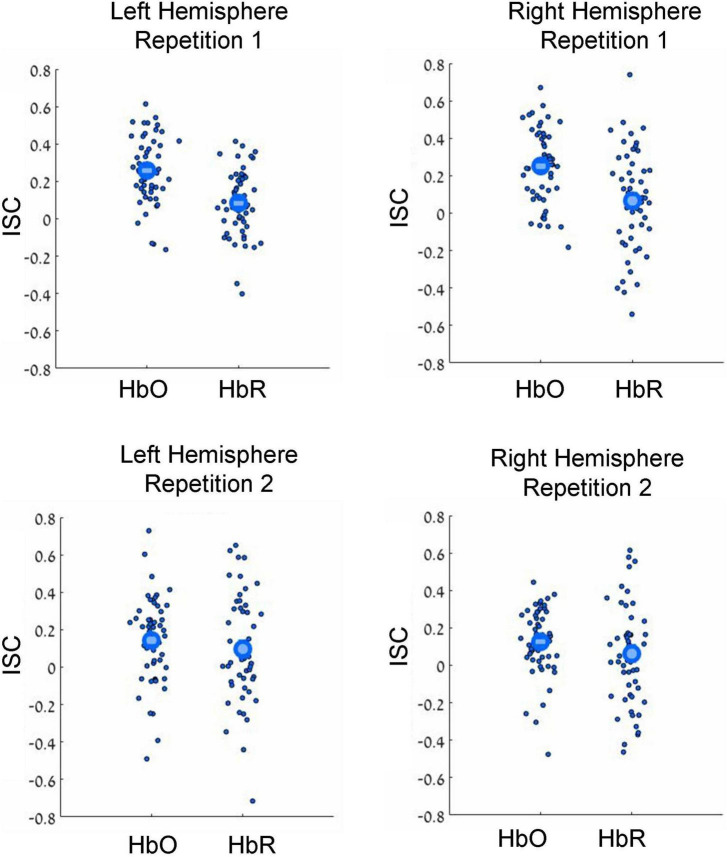
Results of leave-one-subject-out ISC analysis. Results for the first and second movie repetitions are shown in the top and bottom rows, respectively, while results for the left and right hemispheres are shown in the top and bottom columns, respectively. Small dots reflect results for individual participants, while large circles reflect group averages. HbO and HbR stand for oxygenated and deoxygenated signals, respectively. Note beyond-zero correlations (ISC), which were particularly high in the HbO channels during the first movie repetition. Note the higher ISC in the HbO compared to HbR channels.

**FIGURE 3:**
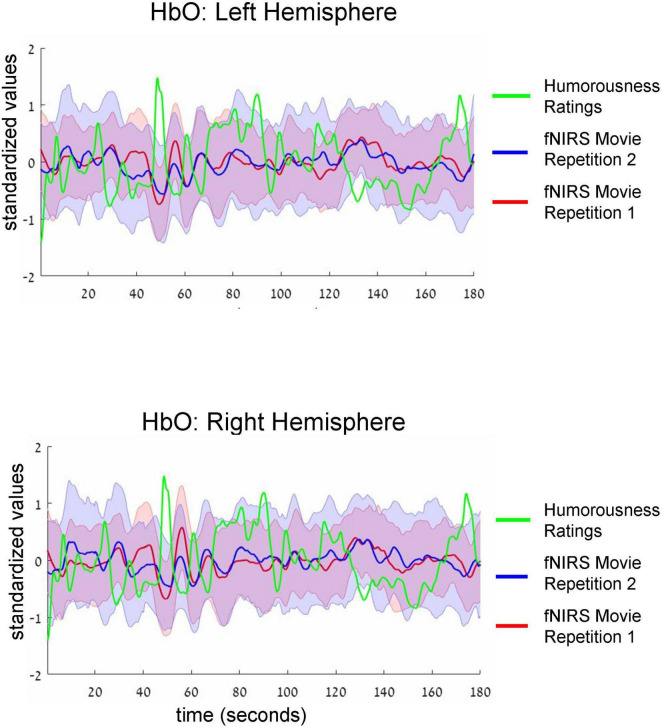
Group-average time-courses of HbO fNIRS neural signals (i.e., two movie repetitions) and the ratings of humorousness of an independent group of participants. Top and bottom plots show fNIRS signals in the left and right hemispheres, respectively. Shadows around the fNIRS time-courses reflect standard error. For clarity of the figure, we do not show the standard deviation of the ratings of humorousness. Note negative correlation (i.e., anti-correlation) between neural signals and the ratings of humorousness. In addition, there is a correlation between the fNIRS signals of two repetitions of the movie.

**FIGURE 4:**
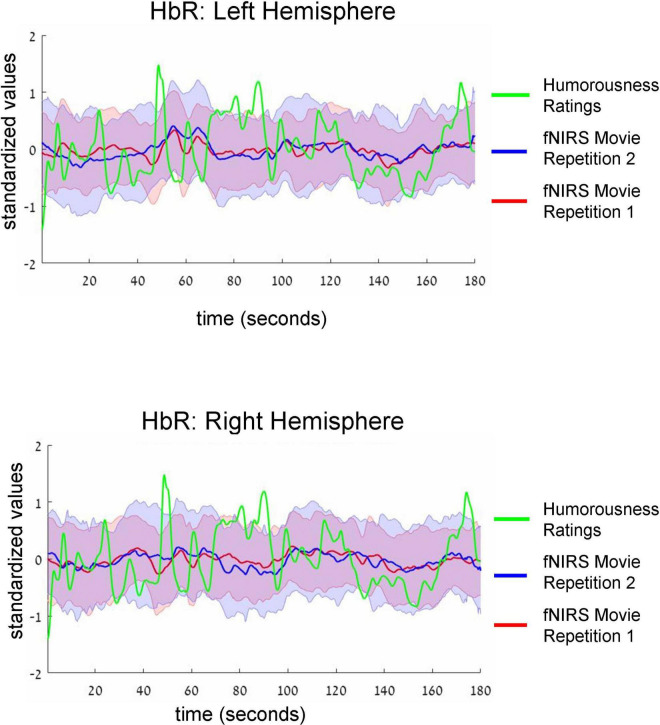
Group-average time-courses of HbR fNIRS neural data (i.e., two movie repetitions) and the ratings of humorousness of an independent group of participants. The same convention as in Figure 3 is used. There was no correlation between neural signals and ratings of humorousness.

## Results

After completing watching two 3-min repetitions of the movie, participants answered a behavioral questionnaire. The first two multiple-choice questions focused on understanding the movie’s content. The percentage of participants that answered these questions correctly was 100 and 98.1%, respectively, indicating that the participants watched and followed the plot of the movie. The two additional questions [Likert scale (1: minimum; 5: maximum)] verified the experiences of the participants while watching the movie. The first question was “To what extent did you have thoughts unrelated to the movie (e.g., personal thoughts)?” The average result was 2.06 (SD: 1.02), indicating that on average the participants concentrated on watching the movie. The second question was “To what extent did you like the movie?” The average result was 3.86 (SD: 0.93), indicating that on average the participants liked the movie.

During the movie, we conducted fNIRS imaging of the bilateral prefrontal cortex. For each hemisphere, a light receiver was surrounded by four emitters (Figure 1), resulting in four oxyhemoglobin (HbO) and four deoxyhemoglobin (HbR) channels (i.e., signals and time-courses) for each hemisphere. It is well established that the signals of proximate fNIRS channels are not independent, (e.g., Niu and He, 2014; Novi et al., 2016; Li et al., 2018; Yücel et al., 2021). In line with this knowledge, in our experiment the average correlation between four prefrontal channels was also high: HbO right hemisphere (Pearson *R*: 0.76, SD: 0.06), HbO left hemisphere (*R*: 0.75, SD: 0.05), HbR right hemisphere (*R*: 0.54, SD: 0.07) and HbR left hemisphere (*R*: 0.56, SD: 0.06). Accordingly, for our analyses, we averaged the signals of four channels, resulting in one oxyhemoglobin (HbO) and one deoxyhemoglobin (HbR) time-course per hemisphere.

In our first analysis, we asked whether activity recorded in the prefrontal cortex while watching the movie correlated across participants. For that we conducted two commonly used ISC analyses (Nastase et al., 2019): the leave-one-subject-out ISC, and the pair-wise ISC analysis (for all methodological details, see “Materials and methods”). Each of these analyses was conducted separately for each of two repetitions of a movie. The results of leave-one-subject-out analysis are shown in Figure 2 and the statistical results are in Table 1 (fourth column). We found beyond-zero correlation for most channels, while the correlation was higher for the HbO, especially in the first repetition. To test for potential statistical differences between movie repetitions, channel types, and hemispheres, we conducted a three-way repeated-measures ANOVA. We found a highly significant main effect of channel type [*F*(52,1) = 25.4, *p* < 0.001, η^2^ = 0.08], significant interaction between movie repetition and channel type [*F*(52,1) = 6.1, *p* = 0.017, η^2^ = 0.022] and relatively high, but not significant main effect of movie repetition [*F*(52,1) = 3.54, *p* = 0.065, η^2^ = 0.02]. No other effects were significant [*F*(52,1) < 1.65]. *Post hoc* paired, two-tailed *t*-tests revealed significantly higher ISC in HbO compared to the HbR channel for the first repetition [left hemisphere: *t*(52) = 5.2, *p* < 0.001, Cohen’s *d* = 0.71; right hemisphere: *t*(52) = 4.06, *p* < 0.001, Cohen’s *d* = 0.56] and higher, but not significantly higher ISC in HbO compared to the HbR channel in the second repetition [left hemisphere: *t*(52) = 1.27, *p* = 0.21, Cohen’s *d* = 0.17; right hemisphere: *t*(52) = 1.56, *p* = 0.11, Cohen’s *d* = 0.21]. There was also higher ISC for the first compared to the second repetition in the HbO [left hemisphere: *t*(52) = 3.17, *p* = 0.003, Cohen’s *d* = 0.44; right hemisphere: *t*(52) = 3.56, *p* = 0.001, Cohen’s *d* = 0.49], but not in the HbR [*t*(52) < 1]. In addition, we repeated our analysis using a pair-wise ISC approach (Table 1, last column). In line with the suggestion of Nastase et al. (2019), the correlation values we obtained in the leave-one-subject-out analysis were much higher than in the pair-wise correlation analysis. Critically, the qualitative effects in the two analyses were very similar, while the highest and most reliable correlation was in the first repetition of the HbO. Statistically, a three-way repeated-measure ANOVA with movie repetitions, channel types, and hemispheres revealed a highly significant main effect of channel type [*F*(1377,1) = 72.61, *p* < 0.001, η^2^ = 0.05], significant interaction between movie repetition and channel type [*F*(1377,1) = 22.055, *p* < 0.001, η^2^ = 0.016], and significant main effect of movie repetition [*F*(1377,1) = 16.464, *p* < 0.001, η^2^ = 0.012]. No other effects were significant [*F*(1377,1) < 2.654]. *Post hoc* paired, two-tailed *t*-tests revealed significantly higher ISC in HbO compared to the HbR channel for the first repetition [left hemisphere: *t*(1377) = 7.72, *p* < 0.001, Cohen’s *d* = 0.21; right hemisphere: *t*(1377) = 7.91, *p* < 0.001, Cohen’s *d* = 0.21]. For the second repetition, ISC in HbO compared to the HbR channel was also higher, but the effects were much weaker [left hemisphere: *t*(1377) = 1.96, *p* = 0.05, Cohen’s *d* = 0.05; right hemisphere: (1377) = 2.47, *p* = 0.013, Cohen’s *d* = 0.07]. There was also higher ISC for the first compared to the second repetition in the HbO [left hemisphere: *t*(1377) = 5.97, *p* < 0.001, Cohen’s *d* = 0.16; right hemisphere: *t*(1377) = 6.44, *p* < 0.001, Cohen’s *d* = 0.17], but not in the HbR [*t*(1377) < 1]. Overall, the main observations were: (a) Significant beyond-zero ISC was found for HbO channels, while the effects were particularly strong in the first movie repetition. (b) In the HbR, the ISC was weaker than in the HbO channels, while beyond-zero significance was found only in the leave-one-subject-out analysis.

**TABLE 1:** Intersubject correlation (ISC) results for two repetitions of the movie bilateral oxygenated (HbO) and deoxygenated (HbR) fNIRS channels.

Movie repetition ID	Channel type	Hemisphere	Leave-one-subject-out ISC analysis	Pair-wise ISC analysis
1	HbO	Left	Mean = 0.26, SEM = 0.02,	Mean = 0.08, SEM = 0.005,
			*t*(52) = 10.5, *p* < 0.001, Cohen’s *d* = 1.44, perm. *p* < 0.001	*t*(1377) = 14.9, *p* < 0.001, Cohen’s *d* = 0.4, perm. *p* < 0.001
1	HbO	Right	Mean = 0.25, SEM = 0.03,	Mean = 0.075, SEM = 0.005,
			*t*(52) = 9.2, *p* < 0.001, Cohen’s *d* = 1.26, perm. *p* < 0.001	*t*(1377) = 14.1, *p* < 0.001, Cohen’s *d* = 0.37, perm. *p* < 0.001
1	HbR	Left	Mean = 0.08, SEM = 0.02,	Mean = 0.02, SEM = 0.007,
			*t*(52) = 3.4, *p* = 0.001, Cohen’s *d* = 0.47, perm. *p* < 0.001	*t*(1377) = 2.4, *p* = 0.16, Cohen’s *d* = 0.06, perm. *p* = 0.14
1	HbR	Right	Mean = 0.07, SEM = 0.04,	Mean = 0.01, SEM = 0.007,
			*t*(52) = 1.8, *p* = 0.076, Cohen’s *d* = 0.25, perm. *p* < 0.001	*t*(1377) = 1.15, *p* = 0.24, Cohen’s *d* = 0.03, perm. *p* = 0.36
2	HbO	Left	Mean = 0.14, SEM = 0.03,	Mean = 0.03, SEM = 0.006,
			*t*(52) = 4.5, *p* < 0.001, Cohen’s *d* = 0.62, perm. *p* < 0.001	*t*(1377) = 5.28, *p* < 0.001, Cohen’s *d* = 0.14, perm. *p* < 0.014
2	HbO	Right	Mean = 0.13, SEM = 0.03,	Mean = 0.03, SEM = 0.005,
			*t*(52) = 5, *p* < 0.001, Cohen’s *d* = 0.69, perm. *p* < 0.001	*t*(1377) = 5.03, *p* < 0.001, Cohen’s *d* = 0.13, perm. *p* = 0.012
2	HbR	Left	Mean = 0.1, SEM = 0.04,	Mean = 0.014, SEM = 0.007,
			*t*(52) = 2.5, *p* = 0.017, Cohen’s *d* = 0.34, perm. *p* = 0.27	*t*(1377) = 2.2, *p* = 0.027, Cohen’s *d* = 0.05, perm. *p* = 0.39
2	HbR	Right	Mean = 0.06, SEM = 0.04,	Mean = 0.01, SEM = 0.006,
			*t*(52) = 1.6, *p* = 0.117, Cohen’s *d* = 0.22, perm. *p* = 0.16	*t*(1377) = 1.07, *p* = 0.28, Cohen’s *d* = 0.03, perm. *p* = 0.22

The fourth and fifth columns contain the results of leave-one-subject-out ISC and pair-wise ISC analyses, respectively. Note the beyond-zero correlations (ISC), which were particularly high in the HbO channels during the first movie repetition.

What can explain lower ISC during the second movie repetition? While various potential explanations can be proposed, one option is that the participants during the second repetition had more artifacts due to motion because they were less focused on the movie. To test the latter potential explanation, for each of the channels we compared between two repetitions the number of excluded samples at the stage of preprocessing between the two repetitions. Indeed, as the results in Table 2 demonstrate, the artifacts during the second movie part were much larger, thus potentially accounting for smaller ISC during the second part.

**TABLE 2:** Comparison of artifacts, likely related to motion between two movie repetitions.

Channel type	Hemisphere	Artifact comparison between repetitions
HbO	Left	Mean repetition 1 = 0.014%, SEM repetition 1 = 0.003,
		mean repetition 2 = 0.059%, SEM repetition 2 = 0.008,
		*t*(52) = 4.9, *p* < 0.001, Cohen’s *d* = 0.67
HbO	Right	Mean repetition 1 = 0.015%, SEM repetition 1 = 0.004,
		mean repetition 2 = 0.075%, SEM repetition 2 = 0.01,
		*t*(52) = 5.31, *p* < 0.001, Cohen’s *d* = 0.72
HbR	Left	Mean repetition 1 = 0.023%, SEM repetition 1 = 0.004,
		mean repetition 2 = 0.055%, SEM repetition 2 = 0.008,
		*t*(52) = 3.49, *p* < 0.001, Cohen’s *d* = 0.48
HbR	Right	Mean repetition 1 = 0.024%, SEM repetition 1 = 0.005,
		mean repetition 2 = 0.067%, SEM repetition 2 = 0.01,
		*t*(52) = 3.91, *p* < 0.001, Cohen’s *d* = 0.53

The results show larger artifacts during the second repetition.

Intersubject correlation between participants found in the previous analysis indicated that prefrontal cortex activity was driven at least to some extent by movie stimulus. Next, we asked whether we could find evidence for processing of specific aspect of the movie (i.e., a specific type of cognitive processing). To this extent, in a separate behavioral experiment, an independent group of 18 participants first watched the same movie fragment and then continuously rated the extent to which each point of the movie fragment was funny (for details, see section “Materials and methods”). Agreement between participants measured as an interclass correlation (Koo and Li, 2016; Marron et al., 2020) was high: *R* = 0.82, *F*(899,15283) = 5.45, *p* < 0.001. Our main goal was to test the association (i.e., correlation) between group-level behavioral ratings of humorousness (independent group) and group-level averages of fNIRS activity (our main experiment). The significance of statistical correlation was established using randomized circular time shifts with 10,000 permutations (Nastase et al., 2019; see section “Materials and methods”). In Figure 3, we show averaged, group-level behavioral ratings of humorousness of a movie (green) and group-level average of fNIRS activity for the HbO channels (red: fNIRS time-course of movie repetition 1; blue: fNIRS time-course of movie repetition 2). We can already see by visual inspection an anti-correlation between behavioral ratings of humorousness and fNIRS activity. In addition, we can see that the time-courses of the two movie repetitions are correlated. Statistically, for the first movie repetition there was a strong and highly significant negative correlation between behavioral scores and bilateral fNIRS signals (left hemisphere: *R* = −0.5, *p* < 0.001; right hemisphere: *R* = −0.53, *p* = 0.002). For the second movie repetition, the correlation was weaker and did not reach significance (left hemisphere: *R* = −0.25, *p* = 0.16; right hemisphere: *R* = −0.31, *p* = 0.06). In Figure 4, we show averaged group-level behavioral ratings of humorousness of a movie and group-level averages of fNIRS activity for the HbR channels. We found no reliable correlation between neural signals and behavioral ratings in any of the channels or repetitions (left hemisphere, first repetition: *R* = 0.1, *p* = 0.4; right hemisphere, first repetition: *R* = 0, *p* = 0.99; left hemisphere, second repetition: *R* = 0.02, *p* = 0.99; right hemisphere, second repetition: *R* = −0.18, *p* = 0.25).

We found a negative correlation in the first movie repetition between fNIRS HbO signals and the humorousness of the movie (Figure 3). In a series of control analyses, we were interested to rule out potentially confounding factors and explanations. First, we tested whether the participants’ motion was correlated with the humorousness of the movie. Our experiment did not include the video recording of the participants (see more details in section “Discussion”). Instead, as the proxy measure of participants’ motion, we used the average number of excluded data-points as part of the preprocessing at each point of the movie (see section “Materials and methods” for more details). Critically, we found no reliable correlation between motion time-course and behavioral humorousness ratings in any of the channels and movie repetitions (HbO first repetition, left hemisphere: *R* = 0.01, *p* = 0.89; HbO first repetition, right hemisphere: *R* = −0.02, *p* = 0.95; HbO second repetition, left hemisphere: *R* = −0.24, *p* = 0.29; HbO second repetition, right hemisphere: *R* = −0.08, *p* = 0.65; HbR first repetition, left hemisphere: *R* = 0.18, *p* = 0.25; HbR first repetition, right hemisphere: *R* = 0.22, *p* = 0.27; HbR second repetition, left hemisphere: *R* = −0.27, *p* = 0.18; HbR second repetition, right hemisphere: *R* = 0, *p* = 0.99). Second, to test whether correlation between HbO signals and the humorousness of the movie is related specifically to processing humorous content, we conducted a series of control analyses using various low-level movie features (see section “Materials and methods” for full details). Specifically, we repeated the original correlation analysis between fNIRS signals and ratings of humorousness using partial correlation while controlling for each of the low-level variables. Critically, in all analyses high negative correlation persisted: auditory volume of a movie as a controlling variable (left hemisphere: *R* = −0.51, *p* < 0.001; right hemisphere: *R* = −0.56, *p* < 0.001), brightness of the movie frames as a controlling variable (left hemisphere: *R* = −0.51, *p* = 0.001; right hemisphere: *R* = −0.53, *p* = 0.002), pixel motion between frames as a controlling variable (left hemisphere: *R* = −0.49, *p* = 0.001; right hemisphere: *R* = −0.52, *p* < 0.001), and scene complexity index as a controlling variable (left hemisphere: *R* = −0.49, *p* = 0.007; right hemisphere: *R* = −0.52 *p* = 0.006). In addition, we directly correlated between fNIRS signals and each of the low-level movie features. Critically, no major correlation was found in any of the analyses: fNIRS signal vs. auditory volume of a movie (left hemisphere: *R* = 0.07, *p* = 0.66; right hemisphere: *R* = 0.17, *p* = 0.2), fNIRS signal vs. brightness of the movie frames (left hemisphere: *R* = −0.2, *p* = 0.12; right hemisphere: *R* = −0.2, *p* = 0.07), fNIRS signal vs. pixel motion between frames (left hemisphere: *R* = 0, *p* = 0.88; right hemisphere: *R* = 0, *p* = 0.99), and fNIRS signal vs. scene complexity index (left hemisphere: *R* = −0.28, *p* = 0.08; right hemisphere: *R* = −0.17, *p* = 0.18). Taken together, it is plausible that the correlation between fNIRS HbO signals and the ratings of humorousness of the movie was related to processing humorous content.

Finally, examination of fNIRS time-courses (Figures 3, 4) also permitted us to compare the fNIRS signals across two repetitions of the movie. First, we can clearly see that the SD during the second repetition was higher than during the first repetition (i.e., the blue shadow extends beyond the red shadow), a result in line with higher ISC during the first compared to the second repetition we found previously (Figure 2). Second, and more interestingly, we can see that the mean signals are correlated across two repetitions. Quantitative analysis (Table 2, third column) confirmed this observation by revealing a significant (after multiple-comparison) correlation in the bilateral HbO and the left HbR channels. Interestingly, we found high correlation between two repetitions even in HbR channels despite the fact that the pair-wise ISC in HbR channels was very low. However, it should be kept in mind that the correlation between two repetitions was conducted between means across 53 participants, and signal averaging improves the signal-to-noise ratio (Klein and Barton, 1963; Hieftje, 1972; Hassan and Anwar, 2010). To complement the correlation between the two repetitions of mean signals, we correlated the time-courses of two repetitions for each individual participant and then made a group-level statistical inference. The results of this analysis are shown in Table 2, fourth column. As expected, absolute values of correlation were much lower (below *R* = 0.1) than for correlation of mean signals. Critically, in the bilateral HbO channels, the correlation was significantly above zero, suggesting some neural processing similarly during two movie repetitions in these channels.

## Discussion

In the present study using fNIRS imaging of the prefrontal cortex, we found a positive ISC during a movie. The ISC was particularly high in the HbO channels during the first repetition of the movie segment. In addition, fNIRS neural activity was negatively correlated with ratings of humorousness of the movie obtained in the independent group of participants. Thus, the key outcome of the present study is that the fNIRS in combination with naturalistic paradigms and the ISC might be a sensitive research approach to studying high-level cognitive processing. In addition, our results suggest the potential role of the prefrontal cortex in humor appreciation. We discuss our results in more detail below.

The research using naturalistic stimuli and ISC has been fruitful during the last two decades (Hasson et al., 2004; Honey et al., 2012b; Simony et al., 2016; Yeshurun et al., 2017; Nummenmaa et al., 2018; Nastase et al., 2019; Sonkusare et al., 2019; Redcay and Moraczewski, 2020; Saarimäki, 2021; Zhang F. et al., 2021). Compared to more traditional, non-naturalistic paradigms (e.g., stimuli with strictly controlled timing and static pictures), a naturalistic approach generates more rich real-life and ecological experiences in the laboratory setup. Most of the studies using naturalistic paradigms and the ISC have been conducted using fMRI (Nastase et al., 2019), while fNIRS has almost never been used. Understanding whether fNIRS can be applied with a naturalistic paradigm and the ISC is particularly important because this approach might be useful for experiments with clinical populations and children. That is, naturalistic paradigms are engaging and relatively effortless (Vanderwal et al., 2015); the fNIRS is portable, accessible, and relatively tolerant of head motion (Pinti et al., 2020). For example, a movie paradigm using fMRI has been successful in diagnosing patients with disorder of consciousness (Naci et al., 2014, 2017). However, MRI might not be available in all hospitals, and even if available, transporting a patient to an MRI imaging unit might be logistically complicated (Monti and Schnakers, 2022). The fNIRS, in contrast, is more available and can be used at the bedside, and therefore might be a more advantageous method in a clinical setting.

By recording the activity in the prefrontal cortex using fNIRS, we found higher-than-zero ISC. The result was particularly strong in the bilateral HbO channels during the first movie repetition (see more discussion below). The result was obtained both using pair-wise and leave-one-subject-out ISC analyses, while in accordance with the suggestion of Nastase et al. (2019), the ISC values in the leave-one-subject-out analysis were much higher than in the pair-wise analysis. Critically, the statistical significance was established both using the parametric *t*-test and the non-parametric permutation approach with a random shift of the time-course. Our results are in line with those in the prefrontal cortex of previous fMRI studies with naturalistic stimuli (Wilson et al., 2008; Kauppi et al., 2010; Lerner et al., 2011; Honey et al., 2012a) and one recent fNIRS study with an auditory narrative (Rowland et al., 2018). Significantly beyond zero ISC that we obtained - similarity of brain activity across participants during watching the movie - indicated that neural activity in the prefrontal cortex was at least to some extent related to or driven by the movie stimulus. That is, when no external stimulus is shown, neural activity should not correlate between participants, as is the case with spontaneous activity (Raichle and Snyder, 2007). Note, however, that one needs to be cautious when interpreting the ISC results. That is, while the beyond-zero ISC might reflect similarity across participants in processing movie content, it might also reflect other factors such as synchronization of arousal across participants when watching the movie. Based on the ISC result alone, it is impossible to resolve between alternatives, especially as we recorded only in the prefrontal cortex, but no additional regions. The magnitude of the ISC effects in the HbO were stronger than in the HbR channels, especially for the first repetition of the movie. HbO effects were stronger in several studies with non-naturalistic stimuli (Miyai et al., 2001; Suzuki et al., 2004; Hoshi, 2007; Jiang et al., 2012; Mol et al., 2019; Luke et al., 2021). Thus, our results extend the knowledge of HbO relatively high sensitivity to naturalistic stimuli and the ISC analysis. It is noteworthy that we do not know the exact reason for higher sensitivity of the HbO compared to HbR channels. One possibility is that the HbR channel was more affected by the global signal. Unfortunately, as we explain in detail below, our design and setup did not allow us to remove the global signal, so this possibility could not be tested.

One interesting aspect of our work was the differences at the neural level between the first and second repetition. Specifically, we found higher ISC for the first compared to the second movie repetition (Figure 2). While in the HbO channels the ISC was significantly beyond zero for both repetitions, in the HbR channels there was no reliable ISC during the second repetition. These results were further corroborated by analysis in which we directly correlated time-courses between two repetitions: the reliable correlation between two repetitions was found only for the HbO channels (Table 3). Thus, at least at some level, reproducibility between the two repetitions was achieved for the HbO channels. What might explain the lower effects during the second repetition? It is possible that during the second repetition, the movie was perceived by the participants as less funny. It is also possible that the participants were less engaged during watching the movie segment for the second time (for a similar result in the fMRI study, see: Jääskeläinen et al., 2016). In line with this possibility, there were more fNIRS signal artifacts (presumably motion artifacts) during the second compared to the first movie repetition. Larger artifacts during the second repetition could also have caused lower ISC due to lower signal quality. In this context, it should be noted that our study did not include video monitoring of the participants’ motion - the procedure that would have been more direct and more precise way to obtain information about participants’ motion. Thus, future similar studies should include video monitoring of the participants. Overall, there are several possibilities or combinations of possibilities that can explain lower effects during the second repetition, although a definite conclusion is unlikely to be reached.

**TABLE 3:** Correlation between fNIRS signals of two movie repetitions.

Channel type	Hemisphere	Group-level correlation	Individual-participant correlation
HbO	Left	*R* = 0.65, *p* = 0.002	Mean *R* = 0.082, SEM = 0.027,
			*t*(52) = 2.99, *p* = 0.004, Cohen’s *d* = 0.41
HbO	Right	*R* = 0.55, *p* = 0.004	Mean *R* = 0.067, SEM = 0.028,
			*t*(52) = 2.39, *p* = 0.02, Cohen’s *d* = 0.33
HbR	Left	*R* = 0.58, *p* < 0.006	Mean *R* = 0.066, SEM = 0.041,
			*t*(52) = 1.61, *p* = 0.11, Cohen’s *d* = 0.22
HbR	Right	*R* = 0.55, *p* < 0.028	Mean *R* = 0.014, SEM = 0.039,
			*t*(52) < 1, Cohen’s *d* = 0.05

The third and fourth columns include the results of correlations at the level of mean time-courses (Figures 3, 4) and at the level of individual participants.

The results of the ISC analysis, as we show above, can establish whether there is a synchronous (i.e., correlated) neural activity in the region of interest while watching a movie. However, the ISC cannot provide, at least directly, a cognitive interpretation of the found phenomenon. To address this, we need to relate the neural signal to a behavioral measure. Here, we successfully demonstrated that neural activity recorded using fNIRS correlated with ratings of humorousness of the movie obtained from the independent group of participants. In a control analysis, we showed that motion artifacts of the participants did not correlate with the humorousness ratings (i.e., participants did not move more during the funnier parts of the movie). In addition, there was no correlation between neural activity recorded using fNIRS with low-level properties of a movie such as auditory volume or pixel brightness. Thus, it is likely that correlation between the neural signal and humorousness ratings reflected high-level cognitive processing, specifically humor processing. The implications of this latter result are discussed next. Note that beyond theoretical impact with regard to humor processing, the importance of our result is that we were able to demonstrate sensitivity of fNIRS to reveal neuro-behavioral correlation for the naturalistic paradigms. That is, one early fNIRS study with naturalistic stimuli demonstrated beyond-zero ISC, but it failed to show any correlation between ISC and behavior (Rowland et al., 2018).

Humor is prominent in our lives, while the positive impact of humor spans from improving interpersonal communication (Martin and Ford, 2018) to reducing stress (Abel, 2002) and relieving pain (Weisenberg et al., 1995; Tse et al., 2010). Humor processing has been investigated using a variety of paradigms, including auditory and visual verbal material as well as static images and short movies (for review: Vrticka et al., 2013). Neural substrates of humor have been investigated primarily using fMRI. Interestingly, no clear localization of humor processing has emerged so far, while activations have been found across the whole cerebral cortex (for review: Vrticka et al., 2013). In our study, we used fNIRS in combination with a 3-min comedy movie - an approach that has not been used previously in humor research. The use of the Chaplin movie permitted us to generate an experience that is close to real life. We found a strong negative correlation between HbO signal recorded using fNIRS and ratings of humorousness of the movie. In other words, the prefrontal cortex was deactivated during more humorous content and activated during less (or non-) humorous content. In a series of control analyses, we showed that the effect we reported is unlikely to be explained by non-humorous sensory properties of the movie (auditory volume, image brightness, pixel motion, and scene complexity). A potential explanation for the deactivation of the prefrontal cortex during humor appreciation might be related to disinhibition of the frontal lobes during emotional and humorous experiences (Wild et al., 2006). In other words, in a non-humorous situation, the prefrontal cortex actively inhibits humor-related behavior (e.g., laughter), but during a humorous situation no inhibition is needed, resulting in deactivation of the prefrontal cortex. In support of this view, lesions in the prefrontal cortex in patients might cause involuntary laughter (Zeilig et al., 1996; Mendez et al., 1999) as well as the emergence of joy and mirth (Mendez and Parand, 2020). In an fMRI study with healthy participants using verbal humorous stories, the authors found deactivation of the right dorsolateral inferior frontal gyrus and the right orbital superior frontal gyrus during humor comprehension (Adamczyk et al., 2017). It is noteworthy that only a few fMRI studies have reported deactivations during humor appreciation. But, given a long-standing tradition in fMRI literature of focusing on activations, it is possible that such deactivation loci existed but were not reported. Finally, in two fNIRS studies, a substantial decrease of the HbO signal in the prefrontal cortex was observed as a result of watching an emotional or funny movie (Matsukawa et al., 2017, 2018). Taken together, our results indicate that humor appreciation might be mediated by deactivation of the prefrontal cortex.

An important aspect to take into consideration during analysis of fNIRS data is that the recorded signal is contaminated by various types of physiological noise and participants’ motion (Kirilina et al., 2013; Nguyen et al., 2018; Pinti et al., 2019; Zhang F. et al., 2021). To address this, we applied standard preprocessing steps (i.e., band-passed filtering, temporal smoothing, detrending, and extraction of segments with motion), thus cleaning the signal from artificial activity like heartbeat component and participants’ motion. However, the signal used in our analysis still contained the global (systemic) signal component; therefore, our results can at least to some extent be explained by the global signal component. The physiological nature of the fNIRS global signal is not fully understood (Chen Y. et al., 2020), while a possible cause of the global signal phenomenon is that near-infrared light, by passing through superficial layers of blood vessels in the skin, changes the signal (Zhang et al., 2016). The global signal might also reflect levels of arousal and vigilance (Chen Y. et al., 2020). The global signal might obscure in particular the results of resting-state connectivity analysis (Obrig et al., 2000; White et al., 2009; Duan et al., 2018; Chen Y. et al., 2020). This is because in this analysis, the time-courses of different regions within the same person are correlated, so the presence of a global signal might artificially boost correlation. But the impact of a global signal in the ISC analysis used in the present study is less evident because the correlation is calculated between participants. For example, in the fMRI field, the controversy around the global signal treatment has been central to resting-state connectivity research (Fox et al., 2005; Murphy et al., 2009; Liu et al., 2017), but we are not aware of any fMRI studies that regressed the global signal in the ISC analyses with naturalistic stimuli (Nastase et al., 2019). Critically, it was impossible to regress the global signal in our experimental setup. That is, our experiment did not include short-separation channels (Scholkmann et al., 2014; Abdalmalak et al., 2022; Paranawithana et al., 2022) or additional physiological measurements such as mean arterial pressure (Scholkmann et al., 2013; Caldwell et al., 2016). Accordingly, only the signal processing methods could be potentially applied for removing the global signal (Yücel et al., 2021). But we recorded only in the frontal lobes with a limited number of channels, so it was impossible to calculate whole-brain global signal and subsequently subtract it (Chen Y. et al., 2020; Yücel et al., 2021). The limited number of fNIRS channels also did not permit using methods such as the principal component analysis (Virtanen et al., 2009; Huo et al., 2021). Note that even if some methods like independent component analysis (Stone, 2002) could potentially be applied, there is no way we can identify which component represents a global signal. That is, studies that used task-evoked design estimated whether the evoked component was enhanced after discarding the presumably global signal component (Kohno et al., 2007; Markham et al., 2009; Virtanen et al., 2009). But in our case there are no evoked responses, therefore, we have no way to know which component is a global signal and which is not. The strategies for cleaning global signals were probably best summarized by Yücel et al. (2021): “In the absence of short-separation channel measurements, a large number of channels or additional measurements of systemic physiology can help in cleaning the signal.” Unfortunately, as none of these measures were available in our study, the global signal could not be cleaned. To account for the global signal, feature naturalistic studies should better use montage with a larger number of channels or/and additional physiological measurements.

## Conclusion

To sum up, using naturalistic movie paradigm fNIRS imaging of the prefrontal cortex, we found an ISC, the highest in the HbO channels during the first movie repetition, as well as correlation between neural activity and ratings of humorousness of the movie. Our results thus suggest that fNIRS with naturalistic stimuli might be a potentially powerful research approach.

## Data availability statement

The raw data supporting the conclusions of this article will be made available by the authors upon reasonable request.

## Ethics statement

This study was approved by the Gonda Multidisciplinary Brain Research Center Ethics Committee at Bar-Ilan University, Israel. The patients/participants provided their written informed consent to participate in this study.

## Author contributions

NS, TM, and YC conducted the fNIRS experiment. NS, TM, and VA analyzed the data and wrote the manuscript. All authors contributed to the article and approved the submitted version.
